# Mindfulness and Symptoms of Depression and Anxiety in the General Population: The Mediating Roles of Worry, Rumination, Reappraisal and Suppression

**DOI:** 10.3389/fpsyg.2019.00506

**Published:** 2019-03-08

**Authors:** Fabrice B. R. Parmentier, Mauro García-Toro, Javier García-Campayo, Aina M. Yañez, Pilar Andrés, Margalida Gili

**Affiliations:** ^1^Department of Psychology and Institute of Health Sciences (iUNICS), University of the Balearic Islands, Palma, Spain; ^2^Balearic Islands Health Research Institute (IdISBa), Palma, Spain; ^3^School of Psychology, The University of Western Australia, Perth, WA, Australia; ^4^Department of Psychiatry, Miguel Servet University Hospital, Zaragoza, Spain; ^5^Department of Nursing and Physiotherapy, University of the Balearic Islands, Palma, Spain

**Keywords:** mindfulness, depression, anxiety, emotional regulation, rumination, meditation

## Abstract

The present study examined the effects of mindfulness on depression and anxiety, both direct and indirect through the mediation of four mechanisms of emotional regulation: worry, rumination, reappraisal and suppression. Path analysis was applied to data collected from an international and non-clinical sample of 1151 adults, including both meditators and non-meditators, who completed an online questionnaire battery. Our results show that mindfulness are related to lower levels of depression and anxiety both directly and indirectly. Suppression, reappraisal, worry and rumination all acted as significant mediators of the relationship between mindfulness and depression. A similar picture emerged for the relationship between mindfulness and anxiety, with the difference that suppression was not a mediator. Our data also revealed that the estimated number of hours of mindfulness meditation practice did not affect depression or anxiety directly but did reduce these indirectly by increasing mindfulness. Worry and rumination proved to be the most potent mediating variables. Altogether, our results confirm that emotional regulation plays a significant mediating role between mindfulness and symptoms of depression and anxiety in the general population and suggest that meditation focusing on reducing worry and rumination may be especially useful in reducing the risk of developing clinical depression.

## Introduction

Mindfulness, the process by which one attends to present-moment sensations, thoughts, emotions and experiences in a non-judgmental manner (e.g., Kabat-Zinn et al., 1992; Marlatt and Kristeller, 1999), has been reported to exert beneficial effects on health and well-being, both in non-clinical (Brown and Ryan, 2003; Coffey et al., 2010; Bränström et al., 2011; Tan and Martin, 2016; Demarzo et al., 2017) and clinical (e.g., Sephton et al., 2007; Galante et al., 2013; Kuyken et al., 2015; Crane et al., 2017; Mitchell et al., 2017; Chu et al., 2018) samples. Of particular interest in the present study, mindfulness, whether dispositional or developed through meditation or intervention, has consistently been associated with lower rates of depression and anxiety (Baer, 2006; Carmody and Baer, 2008; Hofmann et al., 2010; Desrosiers et al., 2013; Tran et al., 2014), including in the general population (Freudenthaler et al., 2017). Since depression and anxiety have major impacts on well-being and are two of the most prevalent psychiatric disorders among primary care patients (Ormel et al., 1994), with sub-clinical symptoms also affecting the general population (Lacruz et al., 2016; Wang et al., 2016), understanding the mechanisms through which mindfulness may impact positively on such symptoms commands interest. While the issue has received an increasing amount of attention in recent years, much remains to investigate regarding the mechanisms underpinning this effect. In this study, we explore the relationship between mindfulness and anxiety and depression in a large sample and within a single statistical model, examining the role of a series of mediators (suppression, reappraisal, ruminations, and worry), and assessing for the first time the role of meditation experience as an independent factor.

Some evidence suggests that emotional regulation, the set of strategies and processes that shape the experience and expression of emotions (Gross, 1998), impact on well-being (Gross and John, 2003) and may play a mediating role between mindfulness and depression. On the one hand, links have been reported between mindfulness and emotional regulation in clinical samples (Desrosiers et al., 2013), in meditators (Tran et al., 2014), and in the general population (Freudenthaler et al., 2017). On the other hand, deficits of emotional regulation are frequently observed in patients suffering from clinical depression or anxiety disorders (Abercrombie et al., 1998; Etkin et al., 2004; Garnefski and Kraaij, 2006; Radkovsky et al., 2014). One prevalent and relevant distinction is that between cognitive reappraisal and expressive suppression (Goldin et al., 2008; Cutuli, 2014). Reappraisal is a cognitive strategy modifying emotional responses through a reformulation of the meaning of a situation. Expressive suppression, in contrast, is a strategy aimed at inhibiting behaviors associated with emotional responses (e.g., facial expressions), reducing the expression of emotions but not their experience (Gross, 2002). Expressive suppression is regarded as a maladaptive strategy and is positively correlated with depression and anxiety (Campbell-Sills et al., 2006). Reappraisal, in contrast, is negatively associated with depression and anxiety (Martin and Dahlen, 2005; Desrosiers et al., 2013; Garland et al., 2011; Peh et al., 2017). Importantly, reappraisal is an essential component of mindfulness meditation and interventions (Goleman and Schwartz, 1976; Feldman et al., 2010; Jha et al., 2010), and has been highlighted as one of the mechanisms through which mindfulness works (Hölzel et al., 2011). Expressive suppression, on the other hand, runs contrary to an important aspect of mindfulness, namely the awareness and acceptance of ones’ emotions (Bishop et al., 2004), but fits with the avoidance behavior common in depression and anxiety disorders (e.g., Struijs et al., 2017). In line with these contentions, mindfulness has been found to be positively associated with cognitive reappraisal but negatively so with expressive suppression (Garland et al., 2011; Hölzel et al., 2011; Froeliger et al., 2012; Desrosiers et al., 2013; Brockman et al., 2017).

Apart from cognitive reappraisal and expressive suppression, other emotional regulation mechanisms have been documented, such as rumination and worry. Rumination consists in repetitive and intrusive thoughts about past negative emotional experiences, including their perceived cause and expected consequences. Several studies reported a negative relationship between rumination and mindfulness in community samples (Kumar et al., 2008; Feldman et al., 2010; Raes and Williams, 2010), as well as a reduction of depressive symptoms in individuals with remitted major depressive disorder who underwent mindfulness-based cognitive therapy (Shahar et al., 2010). Rumination is intrinsic to clinical depression (Joormann et al., 2006) and correlates with symptoms of depression and anxiety in community samples (Burwell and Shirk, 2007; Nolen-Hoeksema et al., 2008). Some researchers have suggested that rumination might mediate the negative relationship between mindfulness and depression (Desrosiers et al., 2013; Alleva et al., 2014) or anxiety (Desrosiers et al., 2013).

Worry can be described as “a chain of thoughts and images, negatively affect-laden, and relatively uncontrollable; it represents an attempt to engage in mental problem-solving on an issue of which outcome is uncertain but contains the possibility of one or more negative outcomes” (Borkovec et al., 1983, p. 10). Uncontrollable worry is a core feature of anxiety disorders (Hoyer et al., 2002, 2009) but also affects adults who do not qualify for such diagnostic (Ruscio et al., 2005; Olatunji et al., 2010). Though research on the effect of mindfulness on worry is relatively scarce, recent evidence indicates that worry decreases when dispositional mindfulness increases, and that mindfulness meditation is effective in reducing the negative impact of distressing images in habitual worriers (Verplanken and Fisher, 2014). Furthermore, mindfulness-based interventions appear to reduce worry in individuals with significant anxiety-related distress (Lenze et al., 2014). Both rumination and worry are characterized by their relative uncontrollability and are thought to reflect reduced cognitive control (Raes and Williams, 2010; Beckwé et al., 2014; Hallion et al., 2014). Mindfulness is characterized by the orienting of attention toward the present moment (as opposed to the past, as is characteristic of rumination, or to the future, as is the case of worry), and the non-judgmental awareness of the transient nature of thoughts and emotions.

In sum, evidence suggests that expressive suppression, worry and rumination are negatively associated with mindfulness, while reappraisal exhibit the opposite relationship. Importantly, evidence indicates that the first three contribute to depression and anxiety or their related symptoms, while the fourth reduce such symptoms. In this study, we sought to test the hypothesis that, in the general population, mindfulness might reduce symptoms of depression and anxiety through the reduction of worry, rumination and expressive suppression, and through the enhancement of reappraisal. While some studies reported findings speaking to this issue, these have focused on clinical samples (Desrosiers et al., 2013) or have considered difficulties in emotion regulation but not specific mechanisms of such regulation (Freudenthaler et al., 2017). Past studies are also heterogeneous with respect to their operationalization of mindfulness. Research on mindfulness typically includes three types of studies: studies based on interventions (e.g., the mindfulness-based stress reduction program or breathing exercises), comparisons of meditators and non-meditators, or studies focusing on dispositional mindfulness (but typically not controlling for, or factoring in, the meditation practice (MP) of participants). We would argue that the distinction between dispositional mindfulness and meditation, though not typically addressed in past studies, is an interesting one in relation to depression and anxiety. The distinction between dispositional and cultivated mindfulness (i.e., developed through meditation or mindfulness training) remains debated (Grossman et al., 2004; Rau and Williams, 2016; Tang et al., 2016; Wheeler et al., 2016). While one can score highly on a measure of dispositional mindfulness without practicing mindfulness meditation, evidence suggests that such meditation is associated with greater mindfulness (Shapiro et al., 2011; Soler et al., 2014) and has measurable effects on brain functioning (Tang et al., 2015). Though discussing the distinction between dispositional and cultivated mindfulness falls beyond the scope of our study, we would argue that both mindfulness level and experience of mindfulness meditation should be taken into account and distinguished when examining the impact of mindfulness on depression and anxiety. Indeed, meditation consists in specific practices that some have regarded as specifically promoting emotional regulation, cognitive control or attention (Tang et al., 2016). As such, MP might constitute a specific type of training and install or develop mechanisms absent or less developed in non-meditators.

We present below the results of a questionnaire study administered to a large, non-clinical, and international sample in which we measured dispositional mindfulness and obtained a numerical estimate of the amount of mindfulness practice accumulated by participants, together with measures of depression and anxiety and of four emotional regulation mechanisms: cognitive reappraisal, expressive suppression, rumination and worry. We hypothesized that (1) mindfulness would lead to reduced levels of depression and anxiety; that (2) mechanisms of emotional regulation would mediate that effect; and (3) that mindfulness MP would contribute to mindfulness. Furthermore, we also examined the prediction that mindfulness meditation would affect depression and anxiety on its own right (that is, independently of its effect on mindfulness). Finally, we aimed to compare the relative contributions of the different emotional regulation mechanisms to the relationship between mindfulness and depression/anxiety.

## Materials and Methods

### Participants

A total of 1494 participants took part in the study. After excluding participants who reported having been diagnosed with a psychological or psychiatric disorder (333) and participants whose level of education could not be ascertained with precision (10), our sample included 1151 adults (900 women) aged 18 to 76 (*M* = 36.34, *SD* = 14.34, Min = 18, Max = 76). Among these, 491 reported practicing mindfulness-based meditation (42.7%). All participants were English (*N* = 410) or Spanish speakers (*N* = 741) and completed the questionnaires in their language of preference (English or Spanish). The sample included participants from 39 countries (mostly from Spain, 53%, Canada, 17%, United States, 9%, Argentina, 5%, and United Kingdom, 4%). The participants’ level of education was scored as follows: primary school education (score of 1); secondary school, high school, vocation/technical school (score of 2); some higher education, graduate or university degree (score of 3); post-graduate studies such as MSc, PhD, or professional degree (score of 4). The mean level of education of our participants was 3.01 (*SD* = 0.756, Min = 1, Max = 4).

### Procedure

Between May 2015 and August 2016, participants were recruited through email announcements (locally, nationally and internationally), and advertisements on online experiment websites for a study on mindfulness and attention or attention and distraction (this variation was introduced in order to attract participants with and without experience of MP). Data were collected through an online survey implemented using the survey development environment Qualtrics. Participation was voluntary and unremunerated. Participants did however receive personalized feedback with their results on the various questionnaires used. Participants first answered some demographic questions (age, sex, level of education) before indicating whether they had ever been diagnosed with any psychological or psychiatric disorder, and providing information regarding their history of meditative practice. They then completed the series of questionnaires described below, presented in a random order except for the questionnaire measuring mindfulness, which was always administered first. This study was carried out in accordance with the recommendations of American Psychological Association with written informed consent from all subjects. All subjects gave written informed consent in accordance with the Declaration of Helsinki. The protocol was approved by the Bioethical Committee of the University of the Balearic Islands.

### Measures

#### Mindfulness: Short Five-Facet Mindfulness Questionnaire (FFMQ)

We administered the short form of the FFMQ (Bohlmeijer et al., 2011) consisting of 24 items measuring five facets of mindfulness (Baer et al., 2006): Observe, Describe, Non-judging of Inner Experience, Acting with Awareness, and Non-reactivity to Inner Experience. Participants rated themselves on a 5-point Likert scale (1 = never, 2 = rarely, 3 = sometimes, 4 = often, 5 = always). For the Spanish version, we used the equivalent items from the Spanish adaptation of the FFMQ (Cebolla et al., 2012). Items from all five subscales were used to compute a total mindfulness score. High scores indicate higher levels of mindfulness. The reliability of the FFMQ, measured in our sample was good (Chronbach’s α = 0.884).

#### Depression and Anxiety: Hospital Anxiety and Depression Scale (HADS)

The HADS contains 14 items assessing levels of depression and anxiety (Zigmond and Snaith, 1983). Half the items measure depression (HADS-D), the others measure anxiety (HADS-A). For each item, the participants selected one of four responses (e.g., “I feel as if I am slowed down” with one of the following options: “nearly all the time,” “very often,” “sometimes,” or “not at all”). For the Spanish version, we used the items from the Spanish version of the HADS (De las Cuevas Castresana et al., 1995). Higher scores indicate greater levels of depression. The HADS-D and HADS-A measures exhibited good reliability, Chronbach’s α (measured in our sample) of 0.753 and 0.798, respectively.

#### Rumination: Leuven Adaptation of the Rumination on Sadness Scale (LARSS)

Rumination was measured using the 17-item LARSS (Raes et al., 2008) and our Spanish translation for Spanish speaking participants. This translation was carried out by a bilingual psychologist external to the study. This translated version was then translated back to English by the first author (bilingual English-Spanish) and compared to the original version to ensure its equivalence. Items (e.g., “I keep thinking about my problems to try and examine where things went wrong”) are rated on a five-point scale (not at all to very much). Higher scores indicate higher levels of rumination, Chronbach’s α = 0.957 (measured in our sample).

#### Worry: Penn State Worry Questionnaire (PSWQ)

The PSWQ is a questionnaire measuring the tendency to worry. In this study, we used the 3-item version of the PSWQ (Berle et al., 2011) and the equivalent items from the Spanish version (Sandin et al., 2009). Each item is rated on a 5-point scale (not at all typical to very typical). Higher scores indicate higher levels of worry. The PSWQ’s reliability, measured in our sample, was good, Chronbach’s α = 0.878.

#### Emotional Regulation: Emotion Regulation Questionnaire (ERQ)

We used the ERQ (Gross and John, 2003) and our Spanish translation (following the same procedure as for the LARSS questionnaire) to assess mechanisms of emotional regulation: cognitive reappraisal and emotive suppression. The ERQ includes 10 items divided in two subscales, one measuring reappraisal (ERQ-Reappraisal, 6 items) and one measuring suppression (ERQ-Suppression, 4 items). Each item is rated on a 7-point scale (from strongly disagree to strongly agree). Both sub-scales exhibited good levels of reliability (measured in our sample): Chronbach’s α = 0.856 for ERQ-R, and Chronbach’s α = 0.806 for ERQ-S.

#### History of Meditation

To measure the participants’ practice of mindfulness meditation, we first asked them whether they practiced or had practiced meditation. If they responded positively, we then assessed how frequently and for how long they practiced the following mindfulness-based types of meditation: Concentrative o Sadhana (e.g., breathing, mantras), Vipassana or contemplative, Visualization techniques, observation of body sensations (e.g., body scan, tai chi, yoga), compassion-based meditation, informal practice of mindfulness (e.g., doing daily activities in a mindful way), or other. The latter two were not regarded as evidence of formal MP. For each type of meditative practice, participants were asked (1) how frequent their MP was (daily, 3–4 times a week, once a week, or never); (2) how long they had been practicing since; and (3) how long their meditation sessions typically were. Based on each participant’s answers to these questions, we derived a numerical estimate of the total number of hours of MP using the following formula:

$$\mathrm{MP}=\sum_{t=1}^{5}{\mathrm{wf}}_{t}\;x\;52\;x{\;\mathrm{ny}}_{t}x\frac{d_{t}}{60}$$

In this formula, *t* is the type of meditation (1 to 5; concentrative, Vipassana, Visualization, Body scan and Compassion type meditation), *wf_t_* is the weekly frequency of meditation based on the participants’ report (7 for “daily”; 3.5 for “3 or 4 times a week,” 1 for “once a week,” 0 for “never”), *ny_t_* is the number of years of practice, and *d_t_* is the typical number of minutes of practice per session. For the data analysis, we used the log value of this estimated number of hours of MP.

## Data Analysis

The questionnaires were set up in order to require all items to be completed. As a result, the data set analyzed below contained no missing data. The data were analyzed using Structural Equation Modeling and a confirmatory analysis approach. The analysis was conducted using AMOS v23 (Arbuckle, 2014). Parameter estimation was based on the maximum likelihood method (non-robust) based on the covariance matrix from the data. Model test statistics, direct and indirect effects were tested based on bootstrapped standard errors, with 5000 bootstrap samples, rendering our estimation method non-parametric (Nevitt and Hancock, 2001). Goodness-of-fit was assessed using the RMSEA and CFI indexes. A RMSEA value inferior to 0.06, and a CFI superior to 0.95, were deemed to offer good model fit (Hu and Bentler, 1999). We selected variables based on our initial hypothesis and we selected variables according to their statistical significance and modification indices of the initial model. To compare models, we calculated the difference in Ataike information criterion and tested it using a chi-square test.

## Results

**FIGURE 1:**
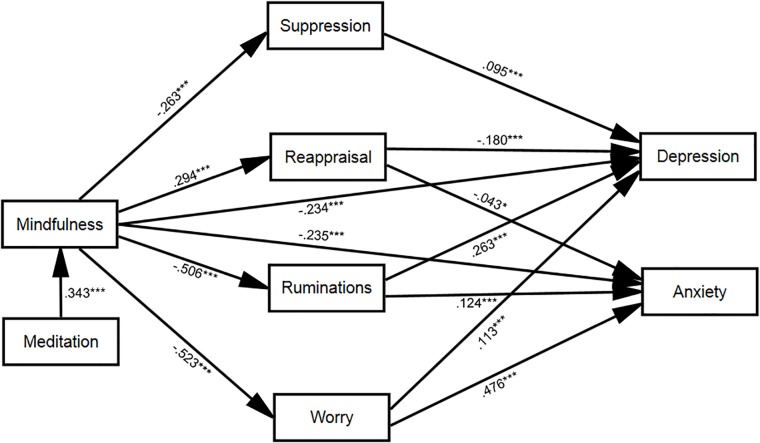
Direct and indirect relationships between mindfulness and depression/anxiety, taking into account the meditating role of emotional regulation (cognitive reappraisal and emotive suppression), rumination and worry. Numbers represent the standardized path coefficients. ^∗^*p* < 0.05, ^∗∗^*p* < 0.01, ^∗∗∗^*p* < 0.001.

The descriptive statistics of the participants’ scores on the questionnaire measures described in the previous section are presented in Table 1. An initial structural equation model was designed in which depression and anxiety were predicted by mindfulness and meditation, four factors were introduced as mediators of the relationship between mindfulness and depression (reappraisal, suppression, ruminations and worry), and the effects of age, gender (1: female, 2: male), level of education (1–4), and language (1: English, 2: Spanish) on depression and anxiety were controlled for. This initial model revealed no association between level of education and depression (*M* = −0.012, *SE* = 0.026, 95% CI: −0.062 to 0.039, *p* = 0.599) or anxiety (*M* = 0.008, *SE* = 0.021, 95% CI: −0.033 to 0.49, *p* = 0.692), nor between gender and anxiety (*M* < 0.001, *SE* = 0.020, 95% CI: −0.040 to 0.038, *p* = 0.989), and no direct association between meditation and depression (*M* = −0.014, *SE* = 0.026, 95% CI: −0.065 to 0.038, *p* = 0.599) or anxiety (*M* = −0.011, *SE* = 0.020, 95% CI: −0.031 to 0.050, *p* = 0.620). Hence these associations were removed and the resulting, adjusted model, evaluated. This adjusted model is illustrated in Figure 1 (for visual clarity, the correlations among error terms and control variables included in the model are not presented in this figure but reported in Table 2). The adjusted model provided a good fit to the data [χ^2^(15) = 24.2, *p* = 0.062, CFI = 0.997, RMSEA = 0.023, 90% CI: 0.000 to 0.039, AIC = 126.176].

**Table 1:** Descriptive characteristics of the participants’ (*N* = 1151) scores on the variables measured in this study.

Variable	*M*	*SD*	Min	Max
Estimated hours of meditation	447.739	1860.453	0	30940
Mindfulness (FFMQ)	79.511	11.475	47	112
Emotive Suppression (ERQ-S)	13.036	5.487	4	28
Cognitive Reappraisal (ERQ-R)	27.817	6.867	6	42
Rumination (LARSS)	41.637	16.296	17	85
Worry (PSWQ)	7.956	3.199	3	15
Depression (HADS-D)	3.904	3.155	0	16
Anxiety (HADS-A)	7.168	3.727	0	19

**Table 2:** Point estimates (M), standard error (SE), lower and upper bounds of the 95% confidence interval (Lower, Upper) and *p*-value (p) of the correlations allowed among error terms (e) and control variables in the adjusted model (these correlations were included in the model based on the modification indices of the initial model).

Variables	*M*	*SE*	Lower	Upper	*p*
Age and Meditation	0.319	0.026	0.268	0.370	<0.001
Age and Gender	0.032	0.029	−0.029	0.087	0.320
Age and e(mindfulness)	0.148	0.027	0.093	0.198	<0.001
Age and e(suppression)	−0.073	0.029	−0.130	−0.016	0.013
Age and e(reappraisal)	−0.097	0.026	−0.150	−0.045	<0.001
Gender and Meditation	0.017	0.031	−0.043	0.078	0.592
Gender and e(worry)	−0.096	0.027	−0.149	−0.043	<0.001
Gender and e(suppression)	0.169	0.028	0.113	0.225	<0.001
Language and Meditation	0.169	0.027	0.114	0.219	<0.001
Language and Gender	0.003	0.029	−0.057	0.056	0.964
Language and Age	0.432	0.027	0.378	0.484	<0.001
Language and e(mindfulness)	0.078	0.028	0.023	0.132	0.005
Language and e(rumination)	0.047	0.026	−0.006	0.096	0.085
Language and e(suppression)	−0.166	0.029	−0.223	−0.112	<0.001
e(depression) and e(anxiety)	0.236	0.034	0.167	0.300	<0.001
e(worry) and e(rumination)	0.381	0.028	0.322	0.433	<0.001
e(worry) and e(reappraisal)	−0.099	0.028	−0.152	−0.041	<0.001
e(suppression) and e(reappraisal)	0.094	0.030	0.034	0.153	0.001
e(suppression) and e(rumination)	0.071	0.029	0.011	0.125	0.019

*These values were obtained using bootstrapping (5000 samples).*

Given the potential theoretical importance of the lack of relationship between MP and depression/anxiety, we also performed partial correlations using bootstrapping (5000 samples) and calculated the Bayes Factor to ascertain whether meditation had any impact on either dependent variable when mindfulness was controlled for: No partial correlation was observed between the amount of MP and anxiety (*r* = −0.043, *SE* = 0.029, 95% CI: −0.100 to 0.014, *p* = 0.146, BF_10_ = 0.131) or between the amount of MP and depression (*r* = −0.003, *SE* = 0.029, 95% CI: −0.059 to 0.054 *p* = 0.930, BF_10_ = 0.056). These results demonstrate that meditation does not affect depression or anxiety when mindfulness is controlled for, and the Bayes Factors provide strong evidence in favor of the absence of correlation.

The statistical details of the path coefficients are presented in Table 3. As visible from Figure 1, mindfulness affected depression and anxiety both directly and indirectly. Cognitive reappraisal, expressive suppression, worry and rumination mediated the relationship between mindfulness and depression: mindfulness increased cognitive reappraisal (negatively correlated with depression) while it decreased expressive suppression, worry and rumination (all positively correlated with depression). The direct effect of mindfulness on depression was significant (*M* = −0.234, *SE* = 0.031, 95% CI: −0.296 to −0.173, *p* < 0.001), as was its indirect effect (*M* = −0.270, *SE* = 0.022, 95% CI: −0.313 to −0.226, *p* < 0.001). A similar picture applied to the relationship between mindfulness and anxiety, with the difference that the meditation included cognitive reappraisal, worry and rumination (not expressive suppression). Mindfulness affected anxiety both directly (*M* = −0.235, *SE* = 0.025, 95% CI: −0.283 to −0.184, *p* < 0.001) and indirectly (*M* = −0.324, *SE* = 0.020, 95% CI: −0.364 to −0.285, *p* < 0.001). Finally, to ascertain the contribution and test the significance of the direct and indirect paths between mindfulness and depression and anxiety, we dropped them from the model (by setting their path weight to zero) and tested the significance of the resulting change in model fit (measured using Akaike’s Information Criterion, AIC, Akaike, 1974) using the chi-square test. As visible from Table 4, all paths made a significant contribution to the adjusted model fit. As reflected by the difference in AIC with and without these paths, the largest contribution to the model fit was provided by the mediation of the worry variable, followed by that of rumination, reappraisal, the direct path from mindfulness to anxiety, the mediation by suppression, and finally the direct path from mindfulness to depression. Finally, to confirm that MP did not affect depression and anxiety directly, we tested a modified model including these direct paths and assessed their coefficients as well as the resulting model fit relative to the model reported above. This analysis confirmed that meditation does not directly affect anxiety (*M* = 0.042, *SE* = 0.58 95%CI: −0.075 to 0.158, *p* = 0.465) or depression (*M* = −0.032, *SE* = 0.064, 95%CI: −0.160 to 0.092, *p* = 0.605). The introduction of these coefficients increased the AIC index by a negligible amount (3.077) and produced no significant difference in model fit [χ^2^(2) = 0.9, *p* = 0.638].

**Table 3:** Point estimates (M), standard error (SE), lower and upper bounds of the 95% confidence interval (Lower, Upper) and *p*-value (p) of the standardized coefficients of the adjusted models depicting the direct and indirect relationship between mindfulness and depression/anxiety, as well as the relationship between meditation practice and mindfulness.

Predictor	*M*	*SE*	Lower	Upper	*p*
**Effect of meditation on mindfulness**					
Meditation	0.343	0.028	0.287	0.395	<0.001
**Effect of mindfulness on mediators**					
Reappraisal	0.294	0.030	0.232	0.352	<0.001
Suppression	−0.263	0.029	−0.321	−0.206	<0.001
Rumination	−0.506	0.024	−0.553	−0.460	<0.001
Worry	−0.523	0.023	−0.566	−0.476	<0.001
**Effect of mediating and control variables on depression**					
Mindfulness	−0.234	0.031	−0.296	−0.173	<0.001
Reappraisal	−0.180	0.026	−0.229	−0.128	<0.001
Suppression	0.095	0.025	0.046	0.145	<0.001
Rumination	0.263	0.032	0.199	0.323	<0.001
Worry	0.113	0.033	0.048	0.177	0.001
Age	0.100	0.026	0.050	0.152	<0.001
Gender	0.054	0.024	0.007	0.102	0.027
Language	−0.060	0.028	−0.115	−0.005	0.032
**Effect of mediating and control variables on anxiety**					
Mindfulness	−0.235	0.026	−0.283	−0.184	<0.001
Reappraisal	−0.043	0.023	−0.088	0.001	0.047
Rumination	0.124	0.026	0.072	0.175	<0.001
Worry	0.476	0.026	0.426	0.527	<0.001
Age	−0.057	0.023	−0.104	−0.012	0.010
Language	−0.126	0.022	−0.167	−0.083	<0.001

*These values were obtained using bootstrapping (5000 samples).*

**Table 4:** Contribution of the direct and indirect paths between mindfulness and depression/anxiety.

	Amputated model		Comparison with full model		
Path removed (Mindfulness to…)	AIC	χ^2^(16)	ΔAIC	χ^2^(1)	*p*-value
Depression	181.101	81.1	54.925	156.901	<0.001
Anxiety	214.073	114.1	87.897	189.873	<0.001
Suppression	201.778	101.8	75.602	177.578	<0.001
Reappraisal	227.969	128	101.793	203.769	<0.001
Rumination	433.408	333.4	307.232	409.208	<0.001
Worry	494.739	394.7	368.563	470.539	<0.001

*This contribution was measured by comparing model fit (AIC) with and without a given path, and examining the significance of resulting change using the chi-square test.*

## Discussion

In the present study, we sought to explore the mechanisms underpinning the relationship between mindfulness and symptoms of depression and anxiety in a general population. More specifically, we sought to determine the extent to which four mechanisms of emotional regulation (cognitive reappraisal, expressive suppression, rumination and worry) mediate this relationship, factoring in the experience of participants with mindfulness meditation. All four emotional regulation mechanisms mediated the relationship between mindfulness and depression, while all but expressive suppression mediated that between mindfulness and anxiety. As expected, mindfulness decreased depression and anxiety by increasing reappraisal (negatively associated with depression and anxiety) and reducing worry, rumination and suppression (negatively associated with depression and, expect for suppression, with anxiety). Worry and rumination proved to be the most potent mediating factors, while suppression and reappraisal played a significant but relatively smaller role. These mediating factors did not entirely account for the relationship between mindfulness and depression or anxiety, however. MP significantly increased mindfulness but had not direct effect on depression or anxiety.

Our findings are consistent with past reports of a negative association between mindfulness on the one hand, and rumination (Kumar et al., 2008; Feldman et al., 2010; Raes and Williams, 2010; Burg and Michalak, 2011; Desrosiers et al., 2013), worry (Desrosiers et al., 2013; Lenze et al., 2014; Verplanken and Fisher, 2014), or suppression (Brockman et al., 2017; Prakash et al., 2017) on the other. Our data are also in line with previous findings of a positive association between mindfulness and reappraisal (Garland et al., 2011; Hölzel et al., 2011; Froeliger et al., 2012; Desrosiers et al., 2013). One original contribution of our study is the comparison of these factors within the same study, however. All four factors acted as independent mediators of the relationship between mindfulness and depression, and three (worry, rumination, and reappraisal) mediated the impact of mindfulness on anxiety. In that respect, our findings differ from those of Desrosiers et al. (2013). Desrosiers et al. examined the relationship between mindfulness and symptoms of depression and anxiety in a sample of 187 participants with mood and anxiety disorders. While they too used reappraisal, rumination and worry as mediating factors in their analysis, they did not include suppression. Instead, they used a measure of non-acceptance of emotional experience, which denotes a propensity to experience negative metareactions to, and avoid the experience of, negative emotions (Gratz and Roemer, 2004; Coffey et al., 2010). Non-acceptance therefore arguably differs from expressive suppression insofar as the latter is a lower-level strategy aimed at inhibiting the behavioral expression of emotional responses. In Desrosiers et al.’s study, worry was the only significant mediator of the relationship between mindfulness and anxiety, and rumination was the only factor mediating the effect of mindfulness on depression. Furthermore, the presence of these mediators eliminated the direct effect of mindfulness on anxiety or depression. In our study, these direct effects remained significant despite the combined effects of all mediating factors. It may be that these discrepancies reflect differences in sample characteristics. Indeed, Desrosiers et al.’s participants presented with mood or anxiety disorders. Our sample, over six times larger, consisted of participants without a history of psychological or psychiatric problems (though this assertion is based on self-reported data in our case, the fact that 333 participants from our initial, unfiltered, sample did report such problems does suggest that this measure was sensitive). Apart from differences with respect to sample characteristics, our study also differs from Desrosiers et al.’s with respect to the measures of depression and anxiety. While Desrosiers et al. used subscales of the Mood and Anxiety Symptoms Questionnaire (Watson et al., 1995), we used the subscales of the HADS (Zigmond and Snaith, 1983). Both scales have been shown to distinguish well between depression and anxiety (Bjelland et al., 2002; Buckby et al., 2007) but, in the absence of any direct comparison between the two, one cannot rule out that they may measure slightly different aspects of these two constructs and, therefore, any comparison between the two studies remains tentative.

In our study, MP correlated positively with mindfulness but did not affect depression or anxiety directly. This suggests that it may not be the actual mental processes exercised by participants during meditation *per se* that help reduce symptoms of depression and anxiety but, rather, the resulting acquisition of aptitudes or skills that affect the way in which participants handle life events and situations more generally. Emotional regulation mechanisms appear to be important variables in that respect. Our analysis indicates that worry and ruminations were the two most potent mediators of the relationship between mindfulness and symptoms of depression and anxiety. Hence, the data suggest that individuals with high dispositional mindfulness are more likely to efficiently regulate their emotions and better handle intrusive thoughts and emotions relating to past or expected events. The practice of mindfulness-based meditation might enhance these abilities. Such contention fits with the prominence, in mindfulness meditation, of paying attention to the present moment (as opposed to directing one’s attention to past or future events). One possibility is that individuals with good meta-cognitive abilities may be more likely to engage in decentering from internal experiences and maintain a certain perspective when faced with events capable of triggering negative emotions. Such abilities may be facilitated and developed though mindfulness-based meditation, possibly through their progressive automatization. Decentering and meta-cognition have recently been argued to be substantial pillars of mindfulness (Shapiro et al., 2006; Jankowski and Holas, 2014; Norman, 2017) and are implicitly referred to in Kabat-Zinn (2003) definition of mindfulness as a state of consciousness that results from being aware of continuous changes in the content of consciousness.

Overall, our results are compatible with the hypothesis that mindfulness, dispositional or enhanced through meditation, can improve well-being by reducing symptoms of depression and anxiety, and that healthy emotional regulation is an important mediator of this effect. Consequently, with respect to strategies for reducing symptoms of depression and anxiety in the general population and help reduce the risk of sub-clinical symptoms converting to clinical disorders, mindfulness-based interventions or meditation exercises focusing specifically on the reduction of worry and rumination may prove especially efficient and beneficial as potential protectors against depression and anxiety in the general population. These conclusions are tentative and should be the focus of future work using complementary methods. While the use of an online survey allowed us to reach a large and more diverse sample of participants than would have been possible in a traditional laboratory-based study, caution must of course be exerted when interpreting the findings since online studies, like laboratory studies, rely on volunteers who may not be entirely representative of the general population (see Birnbaum, 2004; Reips, 2002, for a discussion of the advantages and limitations of internet-based research). While we sought to diversify our sample by portraying our study in two different ways (as a study on mindfulness and attention, or as a study on attention and distraction) and by disseminating it through multiple channels, our study should be interpreted in the context of a larger body of work combining different methodologies and sample types. Finally, it is worth noting that structural equation models are inherently based on correlational data and that causal relationships are therefore derived from a theoretical hypothesis rather than from the data *per se*. We think that the contention we put to the test, namely that mindfulness contributes to a reduction of depression and anxiety and that this effect occurs partly through emotional regulation mechanisms (an hypothesis shared by other researchers, e.g., Desrosiers et al., 2013; Freudenthaler et al., 2017), is a credible one in view of the evidence accumulated so far in the field.

## Data Availability

The datasets generated for this study are available on request to the corresponding author.

## Author Contributions

FP, MG-T, JG-C, PA, and MG contributed to initial research proposal and the acquisition of research funding for this study. FP led the project, programmed the implementation of the online questionnaires, administered the data, and wrote the draft manuscript. MG-T, JG-C, PA, AY, and MG contributed to editing. MG-T and MG carried out the early exploratory analysis of the data. FP and AY finalized the analysis in the form reported in this manuscript.

## Conflict of Interest Statement

The authors declare that the research was conducted in the absence of any commercial or financial relationships that could be construed as a potential conflict of interest.
